# ‘Stopping the start’: support for proposed tobacco control policies – a population-based survey in Great Britain 2021–2023

**DOI:** 10.1136/tc-2023-058571

**Published:** 2024-04-12

**Authors:** Vera Helen Buss, Lion Shahab, Linda Bauld, Loren Kock, Hazel Cheeseman, Jamie Brown

**Affiliations:** 1 Behavioural Science and Health, University College London, London, UK; 2 SPECTRUM Consortium, Edinburgh, UK; 3 Usher Institute and UK Centre for Tobacco and Alcohol Studies, College of Medicine and Veterinary Medicine, University of Edinburgh, Edinburgh, UK; 4 Vermont Center on Behavior and Health, University of Vermont, Burlington, Vermont, USA; 5 Action on Smoking and Health, London, UK

**Keywords:** Public policy, Public opinion, Electronic nicotine delivery devices, End game

## Abstract

**Objectives:**

This study assessed public support for four proposed tobacco control policies in Great Britain: (1) Raising the sales age of tobacco by 1 year every year (Smokefree Generation); (2) Raising the sales age of tobacco from 18 years to 21 years; (3) Providing prescription e-cigarettes as smoking cessation aids to adults who smoke; (4) Restricting e-cigarette advertising to prevent youth uptake.

**Design:**

Repeat cross-sectional population-based survey weighted to match the population of Great Britain.

**Setting:**

The survey was conducted in England, Scotland and Wales in September 2021, October 2022 and October 2023.

**Participants:**

6541 adults living in Great Britain.

**Main outcome measures:**

Support for each policy and year and prevalence ratios (PRs) comparing support between years and subgroups.

**Results:**

The most popular policy each year was restricting e-cigarette advertising (74%/79%/85%), followed by raising the sales age to 21 years (50%/58%/64%), providing prescription e-cigarettes (45%/44%/47%) and Smokefree Generation (34%/44%/49%). The largest increases were for policies about the age of sale (Smokefree Generation: 2021/2022 PR=1.28, 95% CI 1.18 to 1.40, 2022/2023 PR=1.12, 95% CI 1.04 to 1.20; raising the age to 21 years: 2021/2022 PR=1.16, 95% CI 1.09 to 1.23, 2022/2023 PR=1.11, 95% CI 1.05 to 1.17). Only 30% opposed Smokefree Generation in 2023 down from 41% in 2021.

**Conclusions:**

Support for each policy increased each year, except for providing prescription e-cigarettes. Restricting e-cigarette advertising was the most popular policy, while support for age of sale policies, in particular for a Smokefree Generation, grew most.

**Trial registration:**

The study protocol was published on the Open Science Framework (https://osf.io/46z2c/) prior to starting the analysis.

**WHAT IS ALREADY KNOWN ON THIS TOPIC**

- Previous research showed that the British population is more supportive of raising the legal age to 21 years than a Smokefree Generation policy (ban on the sale of tobacco products to everyone born after a certain year).
- It is also known that age, gender and smoking status are associated with level of support for health policies.

**WHAT THIS STUDY ADDS**

- This study showed that between 2021 and 2023 support for policies about raising the age of sale for tobacco and restricting e-cigarette advertising has steadily increased among the British population.
- Even quite novel measures such as making it an offence to sell tobacco products to anyone born after a certain year were supported by half of the population in 2023.

**HOW THIS STUDY MIGHT AFFECT RESEARCH, PRACTICE OR POLICY**

- These results should encourage policymakers to consider bold actions that should pave the way to a smokefree nation and the extent to which leadership can help foster public confidence in proposed measures.

## Introduction

 The UK and Welsh Governments have stated aims for England and Wales to be ‘smokefree’ nations by 2030 (ie, a smoking prevalence below 5%),^1 2^ and the Scottish Government aims for a ‘tobacco-free’ generation in Scotland by 2034.^3^ On current trajectories, further action is likely required to achieve these goals, and different tobacco control policies have been proposed to achieve further reduction in smoking prevalence.^4^,^6^ Additionally, in recent years, rising rates of young people using e-cigarettes have caused concern, with disposable e-cigarettes increasing rapidly in popularity,^7 8^ making this group an important target for newly proposed policies.

The current study focusses on four proposed tobacco control policies: (1) A ban on the sale of tobacco products to everyone born after a certain year from 2030 onwards; (2) Raising the legal age of sale of tobacco products from 18 years to 21 years; (3) Making e-cigarettes available on prescription as a smoking cessation aid for adults who smoke; and (4) Restricting e-cigarette advertising to prevent uptake by young people. In 2022, New Zealand was the first and so far only country that had passed a law to prohibit the sale and supply of smoked tobacco products to anyone born on or after 1 January 2009.^9 10^ The New Zealand Government formed in November 2023 announced—shortly after coming into power—plans to reverse the policy and it has been repealed in February 2024.^10 11^ This policy, often referred to as the ’smokefree generation’ policy, was announced as a future policy for England (to apply to everyone born after 2009) in October 2023 but has not yet been passed into law.^12^ Devolved nations will likely follow England if the law is introduced there. There are currently no definite plans to change the legal age of sale from 18 years to 21 years in any of the British nations. However, it could still be employed as an intermediate measure. The USA passed the law to increase the minimum sales age to 21 years in December 2019 with immediate effect.^13^ Regarding e-cigarettes as smoking cessation tools, the proposed policy would mean that people who smoke could obtain a prescription for government-subsidised e-cigarettes from their general practitioner or a National Health Service stop smoking adviser to help them quit smoking. Under the proposed legislation, e-cigarettes would continue to be available without prescription at an unsubsidised cost. This contrasts with the Australian legislation which only allows access to nicotine-containing e-cigarettes by prescription.^14^ The UK Government announced a national scheme to provide one in five people who smoke with free e-cigarette kits to help them quit,^15^ and is indicative of their objective to maximise the opportunity of e-cigarettes as smoking cessation tools. Recently, the UK Government also announced a consultation to consider different policy options to reduce uptake of e-cigarettes by youth (eg, regulating point of sale displays and e-cigarette packaging and product presentation, preventing industry giving out free e-cigarette samples).^12^

New tobacco control policies have a greater chance of successful implementation if they are accepted by the public and the policymakers are more willing to enact policies when knowing the public supports them.^16^,^18^ Therefore, understanding public attitudes towards proposed tobacco policies can help develop new comprehensive tobacco control strategies that are enacted and complied with by a large portion of the population.^16^ Support may change over time due to media coverage and political debate influencing people’s opinions. Generally, there are differences in support for public health measures by sociodemographic characteristics,^19^ but changes over time may also vary across subgroups. This study addressed the following research questions: (1) What was the level of support for the four proposed tobacco control policies among adults in Great Britain in 2021, 2022 and 2023? (2) Did support differ across nations (England, Scotland, Wales) and between years (2021 vs 2022 and 2022 vs 2023)? (3) Did changes in support differ by age, gender, socioeconomic position (indexed by occupational social grade), presence of children in the household, smoking status and e-cigarette use?

## Methods

### Study design and participants

This study used data collected as part of the Smoking and Alcohol Toolkit Study, a population-based, cross-sectional study with monthly data collection in Great Britain. Approximately 2400 households are selected each month from England, Scotland and Wales through a hybrid strategy of random location and quota sampling. The locations are randomly picked from 227 403 output areas. The areas are stratified by an established geodemographic classification of the population, each including approximately 300 households. Data are collected via telephone by a market research company until quota are fulfilled. The anonymised data are provided to the research team. The survey contains questions about socioeconomic characteristics, smoking and alcohol measures. The policy support questions were included in three survey waves—September 2021, October 2022 and October 2023. Prior to the analysis, the study protocol was published on the Open Science Framework (https://osf.io/46z2c/). When first published, the protocol only mentioned the use of data from 2021 and 2022, but prior to receiving the 2023 data, an amendment was published specifying a comparison between 2021, 2022 and 2023. The raw data used for the analysis are also published on the Open Science Framework.^20^ The paper follows the Strengthening the Reporting of Observational Studies in Epidemiology statement.^21^

### Outcome variables and covariates

The primary outcome measure was level of support for four proposed tobacco control policies. Participants were asked about the extent to which they support these policies. For prevalence estimates, level of support was categorised as ‘supporting’ if ‘strongly support’ or ‘tend to support’ were selected, ‘opposing’ if ‘tend to oppose’ or ‘strongly oppose’ were selected, and ‘indecisive’ if ‘no opinion either way’ or ‘unsure/don’t know’ were selected. For prevalence ratios (PRs), responses of ‘opposing’ and ‘indecisive’ were combined to create a binary outcome variable (‘supportive’ vs ‘not supportive’). The covariates included nation (England, Scotland or Wales), age (18–34 years or 35+ years), gender (women or men), social grade (more advantaged social grades: ABC1, or less advantaged social grades: C2DE),^22^ children in the household (yes or no), smoking status (tobacco smoking, former tobacco smoking or never smoking) and e-cigarette use (yes or no). For gender, descriptive statistics also included the proportion of non-binary participants, but the sample size was too small to include the category in further analyses. More details are provided in the online supplemental material.

### Analysis

The study was based on a complete case analysis. The number of missing values for each variable are listed in the online supplemental table S1. For the first research question, plots were created separately for each policy, showing the weighted proportion and 95% CIs of adults in Great Britain who stated that they supported, opposed or were indecisive about the policy in 2021, 2022 and 2023. For the second research question, plots were created to show for each policy the weighted proportion and 95% CI of adults in England, Scotland and Wales, respectively, who stated supporting, opposing or being indecisive about the policy in each year. Further, weighted PRs and 95% CIs, comparing the proportion of people stating that they supported each policy in 2021 to those in 2022 and in 2022 to those in 2023, across Great Britain and for each nation individually, were calculated. For the third research question, unadjusted and adjusted PRs and their 95% CIs were computed using log-binomial regression and bootstrapping with 2000 replicates^23^ to compare the proportion of people stating that they supported each policy in 2021 to those in 2022, and in 2022 to those in 2023, based on demographic subgroups. These subgroups included (1) People aged 18–34 years versus 35+years; (2) Women versus men; (3) People with more advantaged (ABC1) versus less advantaged (C2DE) social grades; (4) People with versus without children in the household; (5) People who currently versus formerly versus never smoked; and (6) People who used versus did not use e-cigarettes. The online supplemental material includes results from all analyses using unweighted data and adjusted PRs (adjusted using the other variables as covariates). The analysis was conducted in RStudio (V.2022.07.2, R V.4.2.1).

## Results

Complete data were available for 2156 participants from 2021, 2082 participants from 2022, and 2303 participants from 2023 (unweighted). The samples were similar in characteristics, except for a notable increase in e-cigarette use between 2021 and 2023, from 6.7% (95% CI 5.5% to 7.8%) to 13.0% (95% CI 11.3% to 14.8%; online supplemental table S2 and unweighted online supplemental table S3).

### Level of support in Great Britain during 2021–2023

**Figure 1:**
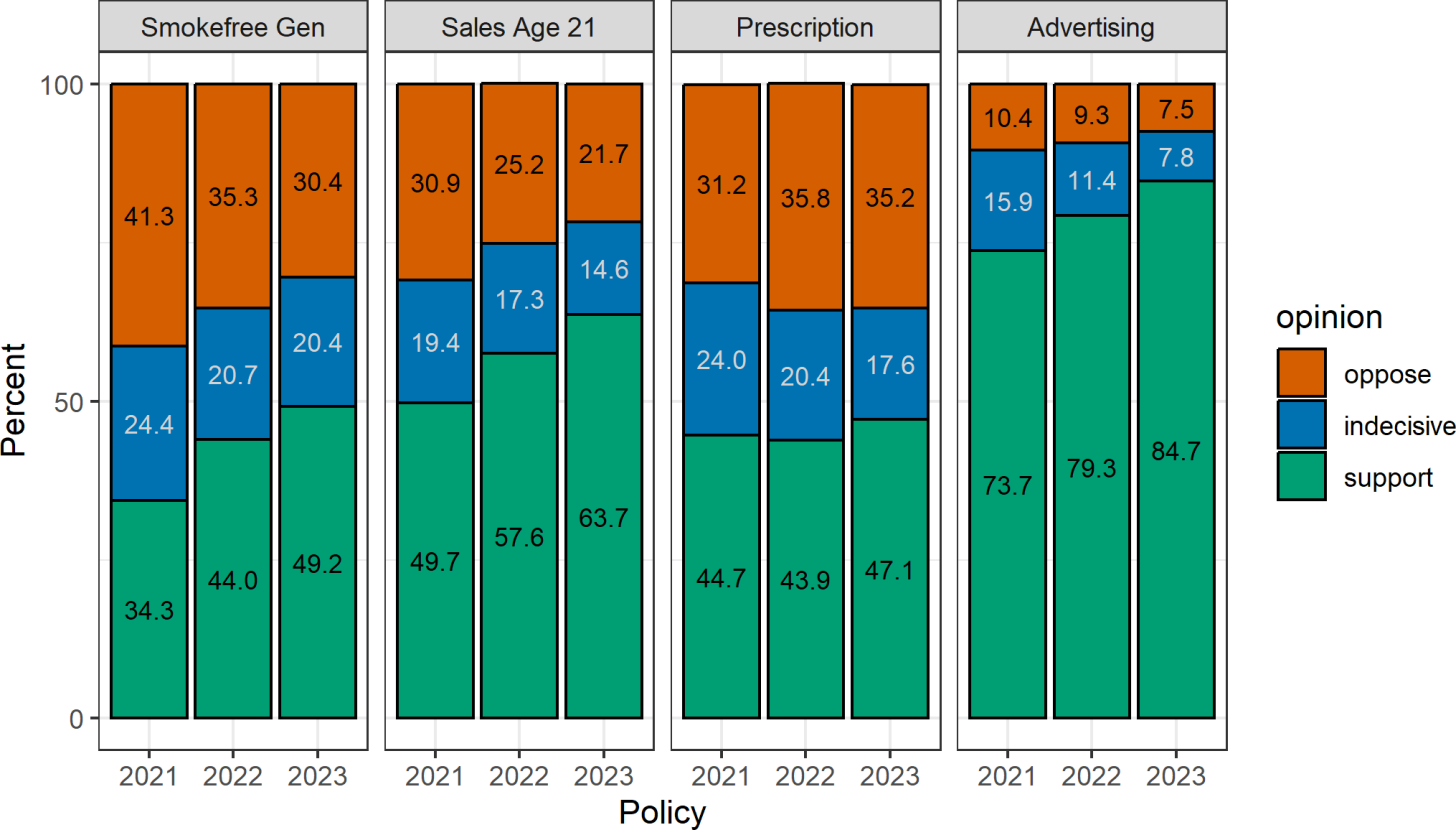
Level of support for each policy in Great Britain during 2021–2023 (weighted). Smokefree Gen, ‘Ban the sale of cigarettes and tobacco products to everyone born after a certain year from 2030 onwards.’; Sales Age 21, ‘Raising the legal age of sale of cigarettes and tobacco from 18 to 21.’; Prescription, ‘Making e-cigarettes available on prescription as a stop-smoking aid for adult smokers.’; Advertising, ‘Restricting e-cigarette advertising to prevent uptake by young people.’.

The highest level of support in all years was for restricting e-cigarette advertising (73.3%, 79.3% and 84.7%), followed by changing the legal age of sale from 18 years to 21 years (49.7%, 57.6% and 63.7%), making e-cigarettes available on prescription for smoking cessation (44.7%, 43.9%, 47.1%), and a sale ban for everyone born after a certain year (34.3%, 44.0% and 49.2%; figure 1, unweighted online supplemental figure S1). For each policy, fewer people were indecisive in the subsequent year compared with the previous. For three policies, opposition declined from year to year. The policy for e-cigarettes on prescription was the only one that slightly more people opposed in 2022 (35.8%) and 2023 (35.2%) than in 2021 (31.2%).

### Differences in level of support between nations and years

**Figure 2:**
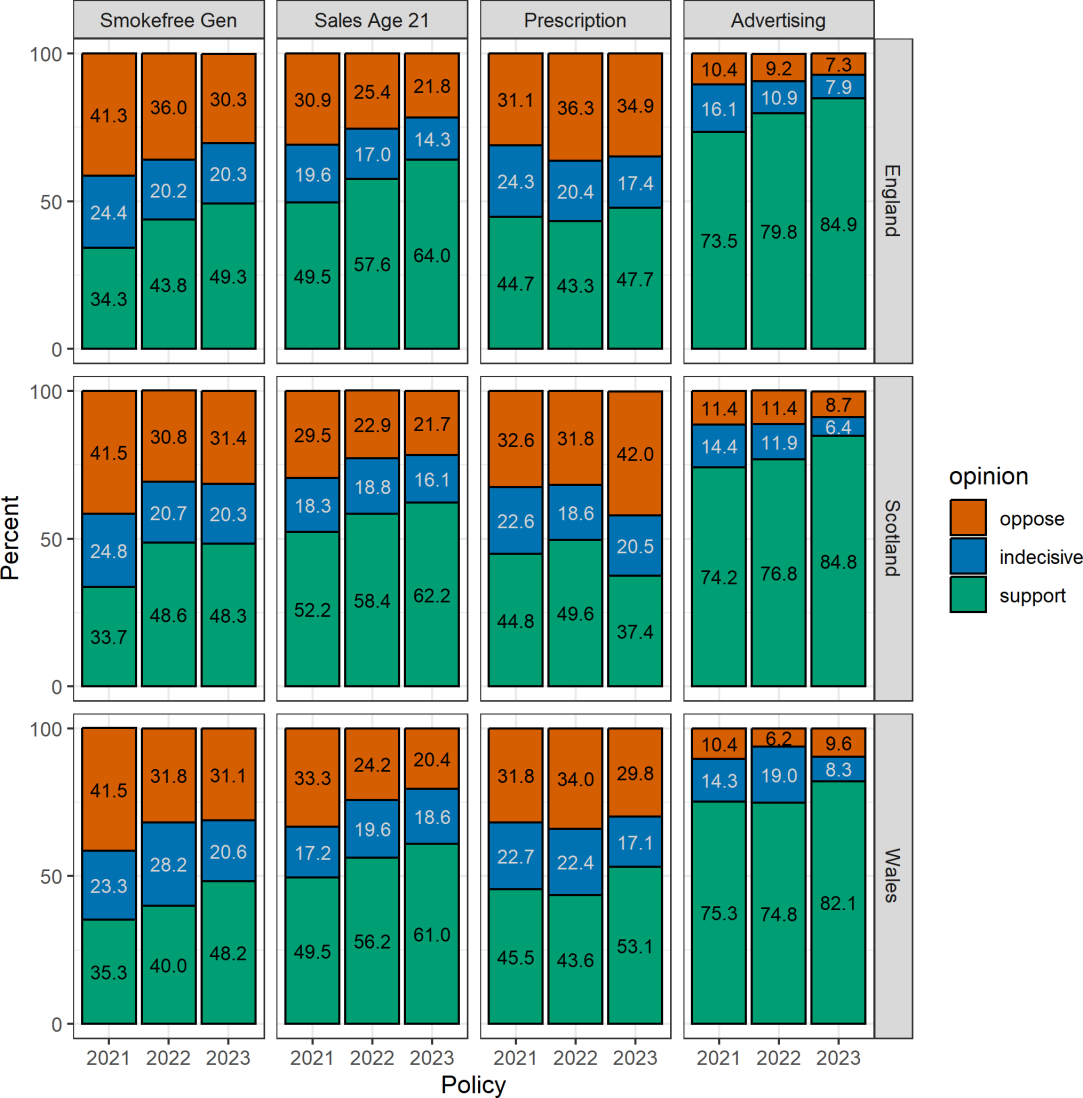
Level of support for each policy in England, Scotland and Wales during 2021 to 2023 (weighted). Smokefree Gen, ‘Ban the sale of cigarettes and tobacco products to everyone born after a certain year from 2030 onwards.’; Sales Age 21, ‘Raising the legal age of sale of cigarettes and tobacco from 18 to 21.’; Prescription, ‘Making e-cigarettes available on prescription as a stop-smoking aid for adult smokers.’; Advertising, ‘Restricting e-cigarette advertising to prevent uptake by young people.’.

The most pronounced difference in level of support between the nations was in relation to making e-cigarettes available on prescription (figure 2, unweighted online supplemental figure S2). While in 2021 and 2022, all nations showed similar levels of support, in 2023, Scotland had a significantly lower level of support (37.4%, 95% CI 32.7% to 42.2%) compared with England (47.7%, 95% CI 45.0% to 50.4%) and Wales (53.1%, 95% CI 46.6% to 59.7%; see online supplemental table S4 and unweighted online supplemental table S5). The difference was due to more people opposing the policy rather than being indecisive in Scotland than in the other nations. Another difference between nations was that Wales tended to have lower levels of support in 2022 than the other two nations, which seemed to be driven by more people being indecisive. However, due to smaller sample sizes in Scotland and Wales, their estimates are subject to higher uncertainty than England. Beyond that, the three nations were relatively comparable.

The level of support for a sale ban for everyone born after a certain year increased significantly in each nation between 2021 and 2022, with the highest increase measured in Scotland, from 33.7% up to 48.6% (table 1 and online supplemental table S4). Between 2022 and 2023, the support for the policy increased further in England and Wales so that all nations had similar levels in 2023, with half being in support of a ban in Great Britain (49.2%, 95% CI 46.8% to 51.6%). From year to year, more people were supportive of a change in the age of sale to 21 years (Great Britain: 2021/2022 PR=1.16, 95% CI 1.09 to 1.23; 2022/2023 PR=1.11, 95% CI 1.05 to 1.17). Similarly, a higher proportion was in favour of restricting e-cigarette advertising in 2022 compared with 2021 (Great Britain: PR=1.08, 95% CI 1.04 to 1.12) and in 2023 compared with 2022 (Great Britain: PR=1.07, 95% CI 1.03 to 1.10). The policy to offer e-cigarettes on prescription to people who want to quit smoking was similarly popular between 2021 and 2022 (Great Britain: PR=0.98, 95% CI 0.91 to 1.06). In 2023, the policy gained support in England (PR=1.10, 95% CI 1.01 to 1.20) and Wales (PR=1.22, 95% CI 0.98 to 1.51), but lost support in Scotland (PR=0.75, 95% CI 0.63 to 0.90).

**Table 1:** Level of support by nation and year, and prevalence ratios (unweighted n=6541, table data weighted)

	Prevalence ratios (95% CI)	
	2021/2022	2022/2023
Ban the sale of cigarettes and tobacco products to everyone born after a certain year		
Great Britain	1.28 (1.18 to 1.40)	1.12 (1.04 to 1.20)
England	1.28 (1.16 to 1.41)	1.13 (1.04 to 1.23)
Scotland	1.44 (1.18 to 1.76)	0.99 (0.85 to 1.17)
Wales	1.13 (0.85 to 1.51)	1.21 (0.95 to 1.53)
Raising the legal age of sale of cigarettes and tobacco from 18 years to 21 years		
Great Britain	1.16 (1.09 to 1.23)	1.11 (1.05 to 1.17)
England	1.16 (1.08 to 1.25)	1.11 (1.04 to 1.18)
Scotland	1.12 (0.97 to 1.29)	1.07 (0.94 to 1.21)
Wales	1.14 (0.92 to 1.41)	1.08 (0.92 to 1.28)
Making e-cigarettes available on prescription as a stop-smoking aid for adult smokers		
Great Britain	0.98 (0.91 to 1.06)	1.07 (0.99 to 1.16)
England	0.97 (0.89 to 1.06)	1.10 (1.01 to 1.20)
Scotland	1.11 (0.93 to 1.32)	0.75 (0.63 to 0.90)
Wales	0.96 (0.75 to 1.23)	1.22 (0.98 to 1.51)
Restricting e-cigarette advertising to prevent uptake by young people		
Great Britain	1.08 (1.04 to 1.12)	1.07 (1.03 to 1.10)
England	1.09 (1.04 to 1.13)	1.06 (1.02 to 1.10)
Scotland	1.03 (0.95 to 1.13)	1.11 (1.02 to 1.19)
Wales	0.99 (0.87 to 1.13)	1.10 (0.98 to 1.23)

### Differences in support by various characteristics

The support for a sales ban for everyone born after a certain year increased similarly across all subgroups between years, except for those who used e-cigarettes, for whom the difference between 2021 and 2022 was uncertain (PR=1.11, 95% CI 0.75 to 1.65), but there was a significant increase in the following year (PR=1.38, 95% CI 1.03 to 1.86; table 2, unweighted online supplemental table S6 and adjusted PRs in online supplemental tables S7 and S8). This increase between 2022 and 2023 brought e-cigarette users closer to the level of support of those who did not use e-cigarettes (40.9% vs 50.5%). Increases in the level of support between the years appeared more pronounced among people currently smoking (2021/2022 PR=1.38, 95% CI 1.00 to 1.89; 2022/2023 PR=1.32, 95% CI 1.03 to 1.72) than those who formerly (2021/2022 PR=1.30, 95% CI 1.09 to 1.55; 2022/2023 PR=1.19, 95% CI 1.02 to 1.39) and never smoked (2021/2022 PR=1.27, 95% CI 1.15 to 1.41; 2022/2023 PR=1.06, 95% CI 0.97 to 1.16), but CIs of the PRs between these subgroups overlap.

**Table 2:** Level of support in Great Britain by subgroups and years, and differences between years in the form of prevalence ratios (unweighted n=6541, table data weighted)

	Support, % (95% CI)			Prevalence ratios (95% CI)	
	2021	2022	2023	2021/2022	2022/2023
Ban the sale of cigarettes and tobacco products to everyone born after a certain year					
Age 18–34 years	34.1	40.5	47.9	1.19 (0.99 to 1.42)	1.18 (1.00 to 1.39)
Age 35+ years	34.5	45.6	49.8	1.32 (1.20 to 1.46)	1.09 (1.00 to 1.19)
Women	34.2	44.1	48.1	1.29 (1.14 to 1.46)	1.09 (0.98 to 1.22)
Men	34.6	44.1	50.4	1.27 (1.13 to 1.44)	1.14 (1.03 to 1.27)
Social grades ABC1	33.6	45.6	51.9	1.36 (1.22 to 1.50)	1.14 (1.05 to 1.23)
Social grades C2DE	35.4	42.2	45.8	1.19 (1.03 to 1.37)	1.09 (0.95 to 1.25)
Children in household	36.7	47.8	53.0	1.30 (1.12 to 1.52)	1.11 (0.97 to 1.27)
No children in household	33.5	42.7	47.8	1.27 (1.15 to 1.41)	1.12 (1.02 to 1.23)
Current smoking	20.7	28.4	37.4	1.38 (1.00 to 1.89)	1.32 (1.03 to 1.72)
Former smoking	31.5	40.9	48.8	1.30 (1.09 to 1.55)	1.19 (1.02 to 1.39)
Never smoking	39.3	49.9	52.9	1.27 (1.15 to 1.41)	1.06 (0.97 to 1.16)
E-cigarette use	26.6	29.5	40.9	1.11 (0.75 to 1.65)	1.38 (1.03 to 1.86)
No e-cigarette use	35.0	46.0	50.5	1.32 (1.21 to 1.44)	1.10 (1.02 to 1.18)
Raising the legal age of sale of cigarettes and tobacco from 18 years to 21 years					
Age 18–34 years	46.6	55.2	58.5	1.18 (1.03 to 1.35)	1.06 (0.94 to 1.20)
Age 35+ years	51.0	58.6	65.7	1.15 (1.07 to 1.23)	1.12 (1.05 to 1.19)
Women	52.8	57.2	65.9	1.08 (1.00 to 1.18)	1.15 (1.06 to 1.25)
Men	46.6	58.0	61.3	1.25 (1.13 to 1.37)	1.06 (0.98 to 1.14)
Social grades ABC1	48.5	57.9	65.2	1.19 (1.11 to 1.29)	1.12 (1.06 to 1.19)
Social grades C2DE	51.4	57.1	61.7	1.11 (1.00 to 1.23)	1.08 (0.98 to 1.19)
Children in household	49.0	64.3	67.9	1.31 (1.17 to 1.47)	1.06 (0.96 to 1.16)
No children in household	50.1	55.0	62.0	1.10 (1.02 to 1.18)	1.13 (1.05 to 1.21)
Current smoking	42.5	51.9	51.0	1.22 (1.01 to 1.48)	0.98 (0.83 to 1.17)
Former smoking	49.3	53.9	64.7	1.09 (0.96 to 1.24)	1.20 (1.07 to 1.34)
Never smoking	51.9	60.8	66.8	1.17 (1.08 to 1.27)	1.10 (1.03 to 1.17)
E-cigarette use	46.8	50.2	56.7	1.07 (0.83 to 1.37)	1.13 (0.92 to 1.38)
No e-cigarette use	50.0	58.6	64.7	1.17 (1.10 to 1.25)	1.10 (1.04 to 1.17)
Making e-cigarettes available on prescription as a stop-smoking aid for adult smokers					
Age 18–34 years	50.0	48.3	47.0	0.97 (0.84 to 1.11)	0.97 (0.84 to 1.12)
Age 35+ years	42.8	41.9	47.1	0.98 (0.89 to 1.07)	1.12 (1.03 to 1.23)
Women	42.4	43.2	46.2	1.02 (0.91 to 1.14)	1.07 (0.96 to 1.19)
Men	47.3	44.4	48.0	0.94 (0.85 to 1.04)	1.08 (0.97 to 1.20)
Social grades ABC1	44.6	42.3	47.9	0.95 (0.86 to 1.04)	1.13 (1.04 to 1.24)
Social grades C2DE	45.1	45.8	46.0	1.02 (0.90 to 1.15)	1.00 (0.88 to 1.14)
Children in household	45.7	40.5	46.3	0.89 (0.76 to 1.04)	1.14 (0.98 to 1.34)
No children in household	44.5	45.1	47.4	1.01 (0.93 to 1.11)	1.05 (0.97 to 1.14)
Current smoking	44.2	46.5	48.1	1.05 (0.87 to 1.28)	1.03 (0.85 to 1.26)
Former smoking	47.1	46.3	47.2	0.98 (0.85 to 1.13)	1.02 (0.88 to 1.18)
Never smoking	43.8	42.0	46.7	0.96 (0.86 to 1.06)	1.11 (1.01 to 1.23)
E-cigarette use	64.8	65.3	62.2	1.01 (0.84 to 1.21)	0.95 (0.81 to 1.12)
No e-cigarette use	43.4	41.0	44.8	0.94 (0.87 to 1.03)	1.09 (1.00 to 1.19)
Restricting e-cigarette advertising to prevent uptake by young people					
Age 18–34 years	73.9	76.0	83.4	1.03 (0.95 to 1.11)	1.10 (1.02 to 1.18)
Age 35+ years	73.7	80.7	85.3	1.10 (1.05 to 1.14)	1.06 (1.02 to 1.10)
Women	74.6	82.4	86.0	1.10 (1.05 to 1.16)	1.04 (1.00 to 1.09)
Men	72.9	76.1	83.4	1.04 (0.99 to 1.11)	1.10 (1.04 to 1.15)
Social grades ABC1	77.0	83.2	88.9	1.08 (1.04 to 1.13)	1.07 (1.04 to 1.10)
Social grades C2DE	69.7	74.3	79.6	1.07 (0.99 to 1.14)	1.07 (1.00 to 1.15)
Children in household	76.6	82.5	87.6	1.08 (1.01 to 1.15)	1.06 (1.00 to 1.13)
No children in household	72.6	78.1	83.7	1.07 (1.03 to 1.12)	1.07 (1.03 to 1.12)
Current smoking	67.6	72.0	76.8	1.06 (0.95 to 1.20)	1.07 (0.96 to 1.19)
Former smoking	72.8	76.3	86.5	1.05 (0.97 to 1.14)	1.13 (1.06 to 1.21)
Never smoking	75.8	82.7	86.3	1.09 (1.04 to 1.14)	1.04 (1.00 to 1.08)
E-cigarette use	72.7	69.1	76.8	0.95 (0.81 to 1.11)	1.11 (0.97 to 1.27)
No e-cigarette use	73.9	80.7	85.9	1.09 (1.05 to 1.13)	1.07 (1.03 to 1.10)

In 2022, most subgroups increased their level of support for changing the age of sale to 21 years, besides people who smoked in the past and those who used e-cigarettes, for whom the increase was uncertain. In 2023, all subgroups increased their support (although uncertainty for a number of these), except people currently smoking (PR=0.98, 95% CI 0.83 to 1.17). There were potential differences by gender and social grade in the extent to which the level of support in sales age changed between 2021 and 2022 (CIs of the PRs between these subgroups overlap). Among men, the level of support increased by a quarter (PR=1.25, 95% CI 1.13 to 1.37), while among women, it only increased by 8% (PR=1.08, 95% CI 1.00 to 1.18). The more advantaged social grade groups (ABC1) had a slightly lower level of support in 2021 (48.5% vs 51.4%) compared with the less advantaged groups (C2DE), but a similar level in 2022 (57.9% vs 57.1%; ABC1: PR=1.19, 95% CI 1.11 to 1.29; C2DE: PR=1.11, 95% CI 1.00 to 1.23). Between 2022 and 2023, the differences between subgroups were less pronounced.

Making e-cigarettes available on prescription as a smoking cessation tool had similar levels of support in 2021 and 2022 across all subgroups. Between 2022 and 2023, the policy gained more support among people aged 35 years and over (PR=1.12, 95% CI 1.03 to 1.23), while it remained similar among younger individuals (PR=0.97, 95% CI 0.84 to 1.12). Those with a more advantaged social grade (ABC1) were more supportive of making e-cigarettes available on prescription in 2023 compared with 2022 (PR=1.13, 95% CI 1.04 to 1.24), while for those of less advantaged social grade (C2DE) the level of support did not change (PR=1.00, 95% CI 0.88 to 1.14). However, CIs of the PRs between these subgroups overlap. On restricting e-cigarette advertising, support became more solidified in 2023 (compared with 2022) across all groups, except potentially among nicotine product users. The level of support increased slightly between 2021 and 2022 among those not using e-cigarettes (PR=1.09, 95% CI 1.05 to 1.13) but remained similar at the same time among people using e-cigarettes (PR=0.95, 95% CI 0.81 to 1.11), although the CIs overlapped. In 2023, e-cigarette users may have reported higher levels of support than in the previous year (PR=1.11, 95% CI 0.97 to 1.27). Overall, the estimates of the adjusted analysis presented in online supplemental table S7 (weighted) and online supplemental table S8 (unweighted) were similar to the unadjusted analysis in table 2 (unweighted online supplemental table S4).

## Discussion

### Summary

The policy of banning the sale of tobacco to everyone born after a certain year received the largest increase in support: by 10 percentage points from 2021 to 2022 and by another 5 percentage points in 2023. Across the years, those who smoke cigarettes and those who do not appeared to have come closer on supporting a smokefree generation policy. For people using e-cigarettes, the level of support did not change between 2021 and 2022 but increased significantly in the following year by almost 40%. From year to year, more people also supported the policy to change the legal age of sale from 18 years to 21 years, with every subgroup increasing their support. Restricting e-cigarette advertising was the policy with the highest level of support in all years, with 85% supporting the policy in 2023, representing an increase of around 11 percentage points from 2021. The policy to offer e-cigarettes on prescription as a smoking cessation aid was similarly popular in all years (45%, 44% and 47%). Overall, the only noteworthy difference between England, Scotland and Wales was that in 2023, the policy to make e-cigarettes available on prescription became less popular in Scotland, while in the other nations it increased in popularity.

### Strengths and limitations

A strength of the study is that it used data from a representative sample with only few missing values. Further, it is the first study assessing changes in support of tobacco control policies in Great Britain by different subgroups. An important limitation is that it is possible that survey participants had not previously heard about the proposed policies and could not make fully informed decisions about whether they support them. This point may be particularly true for the 2021 and 2022 results, prior to government announcements on various tobacco control policies in 2023. The wording of the policy to make e-cigarettes available on prescription might have been ambiguous and therefore may have led some study participants to believe that the policy meant making e-cigarettes exclusively available on prescription, rather than the intended meaning. The statement about the smokefree generation policy included the wording ‘from year 2030 onwards’, but it might have been more meaningful to participants if the birth year of those who would be affected first was made explicit. Also, people may have felt that the year 2030 was relatively far in the future, making the policy less imminent. However, none of those included in the survey would be directly affected by the policy, given it only applied to individuals who are not 18 years old yet.

Further, we reduced the response options for the policy statements from five to two or three, respectively, because we were interested whether people supported the policy (rather than the extent to which individuals supported it). This classification means the analyses may have missed more granular shifts in the extent of support. However, differences in extremity (eg, ‘strongly support’ vs ‘tend to support’) are more related to individual characteristics of people (ie, tendency to select extreme or moderate responses) than to the degree to which they agree with the proposed statement.^24^

### Comparison with existing literature

A previous study focussing on the data of 2021 also assessed the level of support for the two age of sale policies included in the present study.^25^ The support for raising the age of sale to 21 years was positively associated with age (OR=1.06, 95% CI 1.01 to 1.12), and female gender (OR=1.25, 95% CI 1.05 to 1.49), and negatively with current smoking (OR=0.70, 95% CI 0.53 to 0.91, reference: never smoking). For increasing the legal age of sale 1 year every year, the odds of supporting the policy were higher for people from less advantaged social grades C2DE (OR=1.24, 95% CI 1.02 to 1.50) compared with more advantaged social grades ABC1, and lower for people who formerly or currently smoked (OR=0.72, 95% CI 0.58 to 0.89, and OR=0.41, 95% CI 0.41 to 0.56) compared with people who never smoked.^25^ Our study primarily focused on changes in the level of support, so the findings complement each other.

A survey conducted in Great Britain between February and March 2023 by the charity Action on Smoking and Health (ASH) showed that 64% of participants supported raising the sales age from 18 years to 21 years, and 50% supported a sale ban to everyone born after a certain year.^6^ This comparison is interesting considering that data collection for the other survey took place prior to the UK Government’s announcements about potential changes to e-cigarette regulations and the smokefree generation vote,^12^ while our 2023 data were collected around the time of the announcement. In November 2023, ASH asked adults again about their support for a smokefree generation policy and found that 67% supported it in England and 62% in Wales, suggesting support may have further risen.^26 27^ However, there were differences in the framing of the policy: while ASH described the policy in terms of raising the age of sale, our statement described the policy as a ban on sales. These differences may make a difference to how people perceive the policy. Further, the latest ASH Survey emphasised that this was a policy that the UK Government had announced, which may further impact on public acceptability.

The high level of support for restricting e-cigarette advertising in this study might be explainable by many people being concerned about the increasing number of young people using e-cigarettes, which is a topic often featured in the media, and increasingly so since 2021 onwards when disposable e-cigarettes have become more popular.^7 8 28 29^ The marketing of disposable e-cigarettes, such as the product design and packaging, seems to appeal particularly to youth.^30 31^ The policy statement included in the survey specifically mentioned its intention ‘to prevent uptake by young people’. In general, research showed that policies focusing on changing the behaviour of children and young people tend to gain high support.^19^ Framing policies around protection of children against tobacco may also be purposefully used to increase public support.^32^ Looking forward, it will be interesting to see, if some of the policies investigated in the present study are implemented in Great Britain, what impact this will have on public support. Previous research has shown the level of support often increases after a policy has been implemented.^19^

Support for these policies has also been assessed in other countries. For example, an Irish population-based survey from 2022 found that 56% of participants supported a tobacco-free generation policy and 71% the raise of the sales age to 21 years.^33^ A US study assessed the support for raising the age of sale to 21 years prior to its implementation in the USA—between 2014 and 2017, the policy was favoured by around 75% of participants,^34^ showing a higher approval than measured in this study for Great Britain. Another US study using data from 2020 showed that 63% of US American adults supported a ban on tobacco product advertising on social media, 55% a restriction on the location of tobacco product advertising at point of sale, and 50% a ban of tobacco product displays at the checkout counter (e-cigarettes were included in the definition of tobacco products).^35^ These policies are similar to the policy on restricting e-cigarette advertising in the present study. However, differences in support could be due to variations in wording, such as the emphasis on youth e-cigarette use in the current study. In a study in Germany from 2019, 57% of participants supported a complete ban on advertising for e-cigarettes and heated tobacco products.^36^ Again, this figure is substantially lower than what we found but the difference could be partly explained by the significant increase in the use of e-cigarettes since 2019, and that the policy statement in the German survey did not mention young people and referred to a complete ban rather than unspecific restrictions of advertising. Also, Germany has historically had different tobacco control regulations to Great Britain,^37^ so the population in Germany may see the policy as more restrictive than the British population.

It is important to note that high public support does not guarantee that policies will be implemented. Often, when new tobacco control policies have been discussed, the tobacco industry has lobbied against their implementation, arguing that they will lead to policy failure with widely dispersed negative social and economic consequences.^38^ As the example of New Zealand has demonstrated, even if modelling shows the public health benefits of the proposed policy and it is widely supported by the population, policies may still not be implemented due to factors such as industry interference and influence over the political narrative.^39 40^ The availability of a policy champion within government can be an important factor in policy implementation (to counteract other pressures on the government not to act).^41 42^

## Conclusions

While restricting e-cigarette advertising was the most popular policy, restricting the sale of tobacco to younger people has seen increasing support. A higher proportion supported raising the legal age of sale to 21 years than banning it completely to everyone born after a certain year, but the latter was still supported by half of the participants. Given recent announcements in England on one of these policies (sale ban to everyone born after a certain year), our findings could provide further support for policy implementation and also assist policymakers in considering additional future options across the UK. The results may also show policymakers that when they announce that they will proceed with policies, and these policies are therefore more salient to the population, support for the policies may increase.

## Supplementary material

10.1136/tc-2023-058571 online supplemental file 1

## Data Availability

Data are available in a public, open access repository.

## References

[1] Department of Health (2017). Towards a smoke-free generation: a tobacco control plan for England.

[2] Welsh Government (2022). A smoke-free Wales: our long-term tobacco control strategy.

[3] Scottish Government (2018). Raising Scotland’s tobacco-free generation: our tobacco control action plan 2018.

[4] All Party Parlimentary Group on Smoking and Health (2021). Delivering a smokefree 2023: the all parliamentary group on smoking and health recommendations for the tobacco control plan 2021.

[5] Khan J (2022). The Khan review: making smoking obsolete.

[6] Action on Smoking and Health (2023). ASH calls on politicans to listen to the voters and make smoking obsolete.

[7] Tattan-Birch H, Jackson SE, Kock L (2023). Rapid growth in disposable e-cigarette vaping among young adults in Great Britain from 2021 to 2022: a repeat cross-sectional survey. Addiction.

[8] Williams PJ, Cheeseman H, Arnott D (2023). Use of tobacco and e-cigarettes among youth in Great Britain in 2022: analysis of a cross-sectional survey. Tob Induc Dis.

[9] Ministry of Health New Zealand (2023). Smokefree environments and regulated products (smoked tobacco) amendment act Wellington, New Zealand: New Zealand Government. https://www.health.govt.nz/our-work/regulation-health-and-disability-system/smoked-tobacco-products/smokefree-environments-and-regulated-products-smoked-tobacco-amendment-act#:~:text=This%20amendment%20means%20that%20the,or%20after%2C%201%20January%202009.

[10] Mao F (2023). New Zealand smoking ban: health experts critics new government’s shock reversal.

[11] University of Otago (2024). Repeal of smokefree laws means thousands will die, researchers warn Otago, New Zealand. https://www.otago.ac.nz/news/newsroom/repeal-of-smokefree-laws-means-thousands-will-die,-researchers-warn.

[12] Department of Health and Social Care (2023). Stopping the start: our new plan to create a smokefree generation.

[13] US Food and Drug Administration (2021). Tobacco 21. Silver Springs, United States of America: US Food and Drug Administration. https://www.fda.gov/tobacco-products/retail-sales-tobacco-products/tobacco-21.

[14] Department of Health and Aged Care (2020). TGA confirms nicotine e-cigarette access is by prescription only.

[15] Balogun B (2023). The Smokefree ambition for England.

[16] Levy DT, Chaloupka F, Gitchell J (2004). The effects of tobacco control policies on smoking rates: a tobacco control scorecard. J Public Health Manag Pract.

[17] Burstein P (2003). The impact of public opinion on public policy: a review and an agenda. Political Research Quarterly.

[18] Page BI, Shapiro RY (1983). Effects of public opinion on policy. Am Polit Sci Rev.

[19] Diepeveen S, Ling T, Suhrcke M (2013). Public acceptability of government intervention to change health-related behaviours: a systematic review and narrative synthesis. BMC Public Health.

[20] Buss V, Shahab L, Bauld L (2023).

[21] von Elm E, Altman DG, Egger M (2007). The Strengthening the Reporting of Observational Studies in Epidemiology (STROBE) statement: guidelines for reporting observational studies. BMJ.

[22] Collis D (2009). Social Grade: A Classification Tool–Bite Sized Thought Piece.

[23] Efron B, Tibshirani RJ (1994). An Introduction to the Bootstrap.

[24] Peabody D (1962). Two components in bipolar scales: direction and extremeness. Psychol Rev.

[25] Kock L, Shahab L, Moore G (2024). Assessing the profile of support for potential tobacco control policies targeting availability in Great Britain: a cross-sectional population survey. Tob Control.

[26] YouGov (2023). YouGov / ASH survey results. https://d3nkl3psvxxpe9.cloudfront.net/documents/ASH_Results_231117_Eng_W.pdf.

[27] YouGov (2023). YouGov / ASH Wales survey results. https://d3nkl3psvxxpe9.cloudfront.net/documents/ASH_Results_231120_Wales_W.pdf.

[28] East K, Reid JL, Burkhalter R (2022). Exposure to negative news stories about vaping, and harm perceptions of vaping, among youth in England, Canada, and the United States before and after the outbreak of E-cigarette or vaping-associated lung injury (‘EVALI'). Nicotine Tob Res.

[29] Balfour DJK, Benowitz NL, Colby SM (2021). Balancing consideration of the risks and benefits of E-cigarettes. Am J Public Health.

[30] Taylor E, Arnott D, Cheeseman H (2023). Association of fully branded and standardized e-cigarette packaging with interest in trying products among youths and adults in Great Britain. JAMA Netw Open.

[31] Smith MJ, MacKintosh AM, Ford A (2023). Youth’s engagement and perceptions of disposable e-cigarettes: a UK focus group study. BMJ Open.

[32] Kuijpers TG, Willemsen MC, Kunst AE (2018). Public support for tobacco control policies: the role of the protection of children against tobacco. Health Policy.

[33] Cosgrave EJ, Blake M, Murphy E (2024). Is the public ready for a tobacco-free Ireland? A national survey of public knowledge and attitudes to tobacco endgame in Ireland. Tob Control.

[34] Gentzke AS, Glover-Kudon R, Tynan M (2020). Adults' attitudes toward raising the minimum age of sale for tobacco products to 21 years, United States, 2014–2017. Preventive Medicine.

[35] Blake KD, Gaysynsky A, Mayne RG (2022). U.S. public opinion toward policy restrictions to limit tobacco product placement and advertising at point-of-sale and on social media. Preventive Medicine.

[36] Kastaun S, Kotz D (2019). Should advertising for electronic cigarettes and heated tobacco products be banned in Germany? Results of a representative survey (DEBRA study). Bundesgesundheitsblatt Gesundheitsforschung Gesundheitsschutz.

[37] Joossens L, Olefir L, Feliu A (2022). The tobacco control scale 2021 in Europe.

[38] Ulucanlar S, Fooks GJ, Gilmore AB (2016). The policy dystopia model: an interpretive analysis of tobacco industry political activity. PLoS Med.

[39] Hoek J, Edwards R, Waa A (2024). Tobacco industry interference: is the new government meeting its international obligations?. https://www.phcc.org.nz/briefing/tobacco-industry-interference-new-government-meeting-its-international-obligations.

[40] Ait Ouakrim D, Wilson T, Waa A (2024). Tobacco endgame intervention impacts on health gains and Māori:non-Māori health inequity: a simulation study of the Aotearoa/New Zealand tobacco action plan. Tob Control.

[41] Elliott H, Popay J (2000). How are policy makers using evidence? Models of research utilisation and local NHS policy making. J Epidemiol Community Health.

[42] Orem JN, Mafigiri DK, Marchal B (2012). Research, evidence and policymaking: the perspectives of policy actors on improving uptake of evidence in health policy development and implementation in Uganda. BMC Public Health.

