# Supplementary material for: ‘Stopping the start’: support for proposed tobacco control policies – a population-based survey in Great Britain 2021–2023
Source: Tob Control. 2024 Apr 12;34(5):e058571. doi: 10.1136/tc-2023-058571 (PMC12573383; doi:10.1136/tc-2023-058571)
Supplement: online supplemental file 1 [file tc-34-5-s001.pdf]

Supplementary material

Article: "Stopping the start" – support for proposed tobacco control policies: a population-based survey in Great Britain 2021-2023

Table of Contents

Terms used based on definitions from AddictOvocab....1

Outcome variable....1

Covariates....1

Missing values....2

Participant characteristics....2

Unweighted results....2

Adjusted analyses....6

References....7

Terms used based on definitions from AddictOvocab

- *tobacco smoking:* ADDICTO:0000405,[1]
- *former tobacco smoking:* ADDICTO:0000713,[2]
- *never smoking:* ADDICTO:0000779,[3]
- *using e-cigarettes*: ADDICTO:0000664.[4]

Outcome variable

Respondents were asked: "To what extent do you support or oppose this policy suggestion?

1) Ban the sale of cigarettes and tobacco products to everyone born after a certain year from 2030 onwards.
2) Raising the legal age of sale of cigarettes and tobacco from 18 to 21.
3) Making e-cigarettes available on prescription as a stop-smoking aid for adult smokers.
4) Restricting e-cigarette advertising to prevent uptake by young people."

Response options:

i Strongly support
ii Tend to support
iii No opinion either way
iv Tend to oppose
v Strongly oppose
vi Unsure / Don't know

For prevalence estimates, level of support will be categorised as:

- 'Supporting' if response option (i) or (ii) selected,
- 'Opposing' if response option (iv) or (v) selected,
- 'Unsure/No opinion' if response option (iii) or (vi) selected.

For prevalence ratios, responses of 'No' and 'Not sure' will be combined to create a binary outcome variable (supportive vs others).

Covariates

Social grade was included as a measure of socioeconomic position using the National Readership Survey's classification.[5] The category ABC1 comprised high and intermediate managerial, administrative, or professional, supervisory, clerical, and junior managerial, administrative or professional occupations. The less advantaged social grade category, C2DE, included skilled manual workers, semi and unskilled manual workers, state pensioners, casual or lowest grade workers, and unemployed with state benefits only.

Smoking status was determined with the question: "Which of the following best applies to you? (i) I smoke cigarettes (including hand-rolled) every day; (ii) I smoke cigarettes (including hand-rolled), but not every day; (iii) I do not smoke cigarettes at all, but I do smoke tobacco of some kind (e.g. pipe, cigar or shisha); (iv) I have stopped smoking completely in the last year; (v) I stopped smoking completely more than a year ago; (vi.) I have never been a smoker (i.e. smoked for a year or more)." Responses (i), (ii), and (iii) were classified as current tobacco smoking, responses (iv) and (v) as former tobacco smoking, and (vi) as never smoking.

E-cigarette use was assessed with the following questions (with response options including a range of nicotine replacement products): 1) "Which, if any, of the following are you currently using to help you cut down the amount you smoke?"; 2) "Can I check, are you using any of the following either to help you stop smoking, to help you cut down or for any other reason at all?"; 3) "Do you regularly use any of the following in situations when you are not allowed to smoke?"; 4) "Can I check, are you using any of the following for any reason?". People were categorised as using e-cigarettes if they responded to any of the above questions "electronic cigarette" or "Juul".

Missing values

Table S1: Missing values for each variable included in the analysis (N=6609)

	**Missing values, n (%)**
Policy "Ban sale of tobacco products to everyone born after a certain year"	0 (0)
Policy "Raising the legal age of sale of cigarettes and tobacco from 18 to 21"	0 (0)
Policy "Making e-cigarettes available on prescription as a stop-smoking aid"	0 (0)
Policy "Restricting e-cigarette advertising to prevent uptake by young people"	0 (0)
Nation	0 (0)
Age	0 (0)
Gender	26 (0.4)
Social grade	0 (0)
Children in household	0 (0)
Smoking status	42 (0.6)
Using e-cigarettes	0 (0)

Participant characteristics

Table S2: Characteristics of participants (unweighted N=6541, table data weighted)

	**2021**		**2022**		**2023**	
	n	% (95% CI)	n	% (95% CI)	n	% (95% CI)
Age 18-34	607	28.1 (26.0, 30.3)	622	30.0 (27.6, 32.3)	665	28.8 (26.5, 31.1)
Age 35+	1551	71.9 (69.7, 74.0)	1454	70.0 (67.7, 72.4)	1645	71.2 (68.9, 73.5)
Women	1097	50.8 (48.5, 53.1)	1057	51.0 (48.5, 53.4)	1172	50.7 (48.4, 53.1)
Men	1047	48.5 (46.2, 50.8)	1010	48.7 (46.2 51.1)	1125	48.7 (46.3, 51.1)
Non-binary	15	0.7 (0.3, 1.0)	8	0.4 (0.1, 0.7)	13	0.6 (0.3, 0.9)
England	1864	86.4 (85.1, 87.6)	1794	86.4 (85.1, 87.7)	1998	86.5 (85.4, 87.6)
Scotland	189	8.8 (7.8, 9.7)	178	8.6 (7.6, 9.6)	197	8.5 (7.7, 9.4)
Wales	106	4.9 (4.1, 5.6)	104	5.0 (4.2, 5.8)	115	5.0 (4.3, 5.6)
Social grades ABC1	1203	55.7 (53.4, 58.1)	1163	56.0 (53.6, 58.8)	1282	55.5 (53.1, 58.0)
Social grades C2DE	955	44.3 (41.9, 46.6)	913	44.0 (41.4, 46.5)	1028	44.5 (42.0, 46.9)
Children in household	616	28.5 (26.4, 30.7)	578	27.9 (25.6, 30.1)	629	27.2 (25.1, 29.4)
No children in household	1542	71.5 (69.3, 73.6)	1498	72.1 (69.9, 74.4)	1681	72.8 (70.6, 74.9)
Current smoking	321	14.9 (13.2, 16.5)	342	16.5 (14.6, 18.4)	379	16.4 (14.6, 18.3)
Former smoking	592	27.4 (25.4, 29.5)	524	25.2 (23.1, 27.4)	630	27.3 (25.2, 29.4)
Never smoking	1246	57.7 (55.4, 60.0)	1210	58.3 (55.8, 60.7)	1301	56.3 (53.9, 58.7)
E-cigarette use	144	6.7 (5.5, 7.8)	241	11.6 (9.9, 13.3)	300	13.0 (11.3, 14.8)
No e-cigarette use	2014	93.9 (92.2, 94.5)	1835	88.4 (86.7, 90.1)	2010	87.0 (85.2, 88.7)

Unweighted results

Table S3: Characteristics of participants (unweighted N=6541, table data unweighted)

	**Participants n (%)**		
	2021	2022	2023
Age 18-34	527 (24.4)	540 (25.9)	471 (20.5)
Age 35+	1629 (75.6)	1542 (74.1)	1832 (79.5)
Women	1093 (50.7)	1039 (49.9)	1133 (49.2)
Men	1048 (48.6)	1035 (49.7)	1157 (50.2)
Non-binary	15 (0.7)	8 (0.4)	13 (0.6)
England	1621 (75.2)	1547 (74.3)	1586 (68.9)
Scotland	354 (16.4)	348 (16.7)	462 (20.1)
Wales	181 (8.4)	187 (9.0)	255 (11.1)
Social grades ABC1	1376 (63.8)	1453 (69.8)	1563 (67.9)
Social grades C2DE	780 (36.2)	629 (30.2)	740 (32.1)
Children in household	571 (26.5)	558 (26.8)	529 (23.0)
No children in household	1585 (73.5)	1524 (73.2)	1774 (77.0)
Current smoking	306 (14.2)	307 (14.7)	332 (14.4)
Former smoking	611 (28.3)	533 (25.6)	650 (28.2)
Never smoking	1239 (57.5)	1242 (59.7)	1321 (57.4)
E-cigarette use	140 (6.5)	220 (10.6)	233 (10.1)
No e-cigarette use	2016 (93.5)	1862 (89.4)	2070 (89.9)

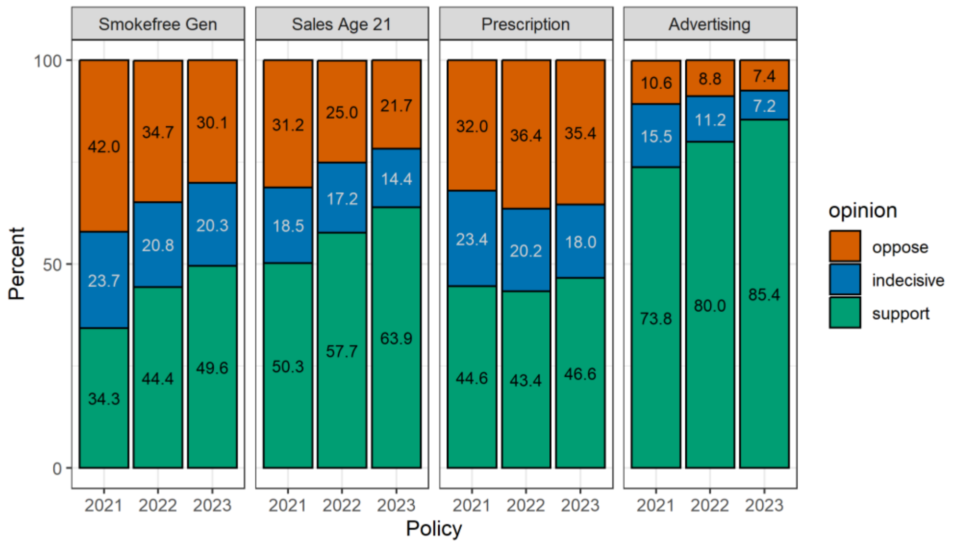

Figure S1: Level of support for each policy in Great Britain in 2021 to 2023 (unweighted). Acronyms: Smokefree Gen, "Ban the sale of cigarettes and tobacco products to everyone born after a certain year from 2030 onwards."; Sales Age 21, "Raising the legal age of sale of cigarettes and tobacco from 18 to 21."; Prescription, "Making e-cigarettes available on prescription as a stop-smoking aid for adult smokers."; Advertising, "Restricting e-cigarette advertising to prevent uptake by young people.".

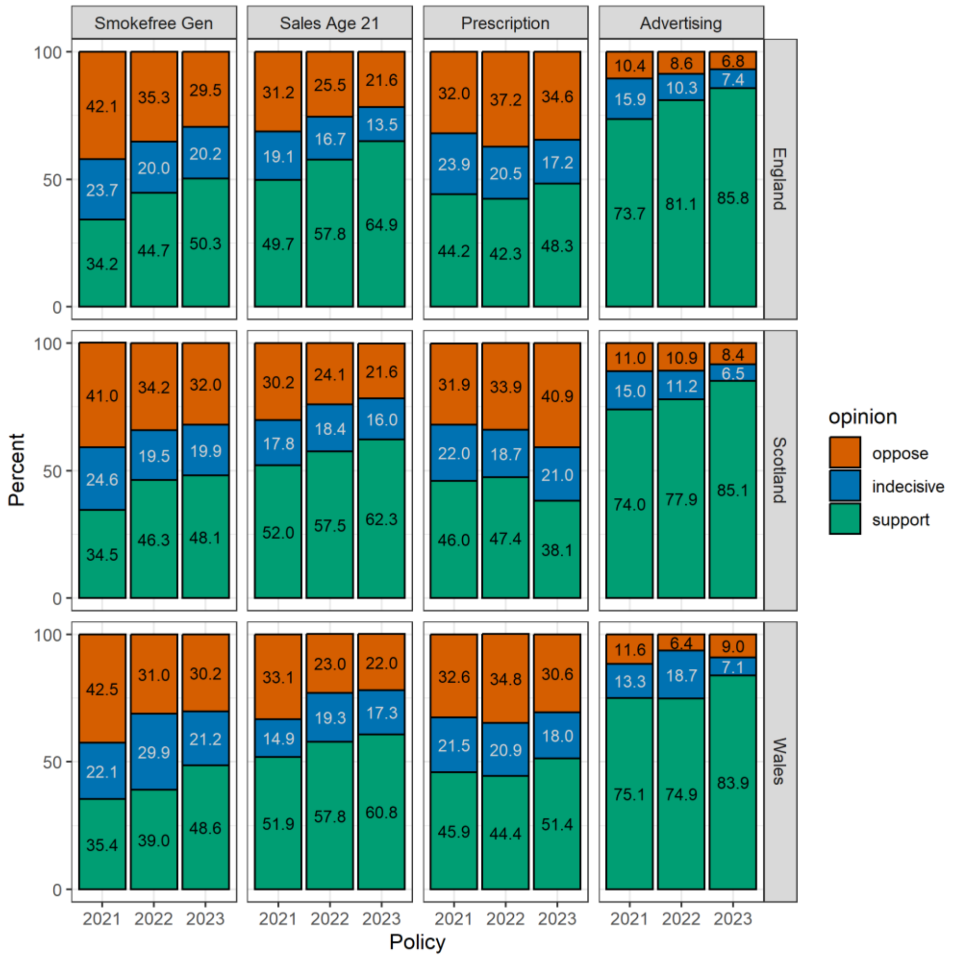

Figure S2: Level of support for each policy in England, Scotland, and Wales in 2021 to 2023 (unweighted). Acronyms: Ban, "Ban the sale of cigarettes and tobacco products to everyone born after a certain year from 2030 onwards."; T21, "Raising the legal age of sale of cigarettes and tobacco from 18 to 21."; ECRx, "Making e-cigarettes available on prescription as a stop-smoking aid for adult smokers."; ECad, "Restricting e-cigarette advertising to prevent uptake by young people.".

Table S4: Weighted level of support by nation and subgroup for each year.

	Support, % (95% CI)		
	2021	2022	2023
**Ban the sale of cigarettes and tobacco products to everyone born after a certain year**			
Great Britain	34.3 (32.1, 36.5)	44.0 (41.6, 46.5)	49.2 (46.8, 51.6)
England	34.3 (31.8, 36.8)	43.8 (41.1, 46.5)	49.3 (46.6, 52.0)
Scotland	33.7 (28.5, 38.9)	48.6 (42.8, 54.4)	48.3 (43.4, 53.2)
Wales	35.3 (27.9, 42.7)	40.0 (32.3, 47.6)	48.2 (41.6, 54.8)
**Raising the legal age of sale of cigarettes and tobacco from 18 to 21**			
Great Britain	49.7 (47.4, 52.0)	57.6 (55.1, 60.0)	63.7 (61.3, 66.0)
England	49.5 (46.8, 52.1)	57.6 (54.8, 60.3)	64.0 (61.3, 66.6)
Scotland	52.2 (46.7, 57.7)	58.4 (52.7, 64.1)	62.2 (57.4, 67.0)
Wales	49.5 (41.7, 57.2)	56.2 (48.4, 64.0)	61.0 (54.6, 67.3)
**Making e-cigarettes available on prescription as a stop-smoking aid for adult smokers**			
Great Britain	44.7 (42.4, 47.0)	43.9 (41.1, 46.3)	47.1 (44.7, 49.5)
England	44.7 (42.1, 47.3)	43.3 (40.6, 46.1)	47.7 (45.0, 50.4)
Scotland	44.8 (39.3, 50.3)	49.6 (43.8, 55.4)	37.4 (32.7, 42.2)
Wales	45.5 (37.8, 53.2)	43.6 (35.9, 51.4)	53.1 (46.6, 59.7)
**Restricting e-cigarette advertising to prevent uptake by young people**			
Great Britain	73.7 (71.6, 75.7)	79.3 (77.3, 81.3)	84.7 (82.9, 86.5)
England	73.5 (71.2, 75.8)	79.8 (77.5, 82.1)	84.9 (82.8, 86.9)
Scotland	74.2 (69.3, 79.1)	76.8 (71.8, 81.7)	84.8 (81.2, 88.4)
Wales	75.3 (68.7, 82.0)	74.8 (67.9, 81.6)	82.1 (76.8, 87.4)

Table S5: Level of support by nation and year, and differences between years in form of prevalence ratios (unweighted, N=6541)

	Support, % (95% CI)			Prevalence ratios (95% CI)	
	2021	2022	2023	2021/2022	2022/2023
**Ban the sale of cigarettes and tobacco products to everyone born after a certain year**					
Great Britain	34.3	44.4	49.6	1.29 (1.22, 1.37)	1.12 (1.05, 1.18)
England	34.2	44.7	49.3	1.31 (1.22, 1.40)	1.13 (1.05, 1.20)
Scotland	34.5	46.3	48.3	1.34 (1.16, 1.53)	1.04 (0.89, 1.19)
Wales	35.4	39.0	48.2	1.10 (0.97, 1.23)	1.25 (1.02, 1.47)
**Raising the legal age of sale of cigarettes and tobacco from 18 to 21**					
Great Britain	50.2	57.7	63.9	1.15 (1.09, 1.20)	1.11 (1.06, 1.15)
England	49.7	57.8	64.0	1.16 (1.10, 1.23)	1.12 (1.07, 1.18)
Scotland	52.0	57.5	62.2	1.11 (0.97, 1.24)	1.08 (0.97, 1.20)
Wales	51.9	57.8	61.0	1.11 (0.98, 1.24)	1.05 (0.90, 1.21)
**Making e-cigarettes available on prescription as a stop-smoking aid for adult smokers**					
Great Britain	44.6	43.4	46.6	0.97 (0.90, 1.04)	1.07 (1.01, 1.14)
England	44.2	42.3	47.7	0.96 (0.88, 1.04)	1.14 (1.06, 1.22)
Scotland	46.0	47.4	37.4	1.03 (0.87, 1.19)	0.80 (0.64, 0.97)
Wales	45.9	44.4	53.1	0.97 (0.84, 1.10)	1.16 (0.96, 1.36)
**Restricting e-cigarette advertising to prevent uptake by young people**					
Great Britain	73.8	80.0	85.4	1.08 (1.04, 1.11)	1.07 (1.04, 1.10)
England	73.7	81.1	84.9	1.10 (1.06, 1.14)	1.06 (1.03, 1.09)
Scotland	74.0	77.9	84.8	1.05 (0.97, 1.14)	1.09 (1.03, 1.16)
Wales	75.1	74.9	82.1	1.00 (0.87, 1.13)	1.12 (1.02, 1.22)

Table S6: Level of support in Great Britain by subgroups and years, and differences between years in form of prevalence ratios (unweighted, N=6541)

	Support, % (95% CI)			Prevalence ratios (95% CI)	
	2021	2022	2023	2021/2022	2022/2023
**Ban the sale of cigarettes and tobacco products to everyone born after a certain year**					
Age 18-34	33.7	41.3	49.1	1.22 (1.04, 1.44)	1.19 (1.03, 1.37)
Age 35+	34.6	45.6	49.8	1.32 (1.21, 1.44)	1.09 (1.02, 1.17)
Women	34.3	44.7	48.5	1.30 (1.17, 1.45)	1.09 (0.99, 1.19)
Men	34.5	44.3	50.7	1.28 (1.15, 1.43)	1.14 (1.04, 1.25)
Social grades ABC1	33.6	45.0	51.7	1.34 (1.22, 1.47)	1.15 (1.07, 1.24)
Social grades C2DE	35.9	43.5	45.3	1.21 (1.06, 1.34)	1.04 (0.92, 1.18)
Children in household	36.8	50.1	55.1	1.36 (1.19, 1.56)	1.10 (0.98, 1.23)
No children in household	33.6	42.5	48.0	1.26 (1.15, 1.39)	1.13 (1.05, 1.22)
Current smoking	21.5	30.3	35.5	1.41 (1.06, 1.86)	1.17 (0.93, 1.47)
Former smoking	32.0	41.7	48.5	1.30 (1.12, 1.52)	1.16 (1.02, 1.33)
Never smoking	38.8	49.2	53.8	1.27 (1.21, 1.33)	1.09 (1.05, 1.14)
E-cigarette use	28.3	30.9	40.7	1.09 (0.78, 1.53)	1.32 (1.02, 1.71)
No e-cigarette use	34.8	46.1	50.7	1.32 (1.22, 1.43)	1.10 (1.03, 1.17)
**Raising the legal age of sale of cigarettes and tobacco from 18 to 21**					
Age 18-34	47.5	57.0	60.3	1.20 (1.07, 1.35)	1.06 (0.95, 1.17)
Age 35+	51.3	58.0	64.8	1.13 (1.06, 1.20)	1.12 (1.06, 1.18)
Women	53.3	59.0	66.5	1.11 (1.03, 1.19)	1.13 (1.06, 1.20)
Men	47.2	56.5	61.3	1.20 (1.10, 1.30)	1.08 (1.01, 1.16)
Social grades ABC1	49.7	58.3	65.3	1.17 (1.10, 1.25)	1.12 (1.06, 1.18)
Social grades C2DE	51.4	56.5	61.0	1.10 (1.00, 1.21)	1.08 (0.99, 1.18)
Children in household	49.4	65.4	69.0	1.32 (1.20, 1.46)	1.06 (0.97, 1.15)
No children in household	50.7	55.0	62.4	1.08 (1.02, 1.16)	1.13 (1.07, 1.20)
Current smoking	43.0	50.5	50.6	1.17 (0.99, 1.39)	1.00 (0.86, 1.16)
Former smoking	49.3	54.7	64.0	1.11 (0.99, 1.24)	1.17 (1.06, 1.29)
Never smoking	52.6	60.9	67.2	1.16 (1.11, 1.21)	1.10 (1.06, 1.15)
E-cigarette use	49.3	49.5	57.1	1.01 (0.81, 1.25)	1.15 (0.97, 1.37)
No e-cigarette use	50.4	58.7	64.6	1.16 (1.10, 1.23)	1.10 (1.05, 1.15)
**Making e-cigarettes available on prescription as a stop-smoking aid for adult smokers**					
Age 18-34	49.4	47.1	46.6	0.95 (0.84, 1.08)	0.99 (0.87, 1.13)
Age 35+	43.2	42.0	46.5	0.97 (0.90, 1.06)	1.11 (1.03, 1.20)
Women	41.9	42.4	45.4	1.01 (0.92, 1.12)	1.07 (0.97, 1.18)
Men	47.6	44.2	47.7	0.93 (0.84, 1.02)	1.08 (0.99, 1.18)
Social grades ABC1	44.3	42.7	42.7	0.96 (0.89, 1.05)	1.10 (1.01, 1.19)
Social grades C2DE	45.3	44.6	44.6	0.98 (0.88, 1.10)	1.03 (0.91, 1.15)
Children in household	45.7	42.5	47.0	0.93 (0.82, 1.06)	1.11 (0.97, 1.26)
No children in household	44.3	43.6	46.5	0.98 (0.91, 1.07)	1.07 (0.99, 1.15)
Current smoking	43.4	46.9	46.4	1.08 (0.90, 1.29)	0.99 (0.83, 1.17)
Former smoking	47.5	45.7	47.3	0.96 (0.84, 1.09)	1.04 (0.91, 1.17)
Never smoking	43.6	41.4	46.2	0.95 (0.91, 0.99)	1.12 (1.07, 1.16)
E-cigarette use	67.4	63.6	63.6	0.94 (0.81, 1.10)	1.00 (0.87, 1.15)
No e-cigarette use	43.1	40.9	44.6	0.95 (0.88, 1.02)	1.09 (1.02, 1.17)
**Restricting e-cigarette advertising to prevent uptake by young people**					
Age 18-34	73.6	76.5	84.3	1.04 (0.97, 1.12)	1.10 (1.04, 1.17)
Age 35+	74.0	81.2	85.7	1.10 (1.06, 1.14)	1.06 (1.02, 1.09)
Women	75.0	83.1	86.3	1.11 (1.06, 1.16)	1.04 (1.00, 1.08)
Men	72.8	77.0	84.6	1.06 (1.01, 1.11)	1.10 (1.05, 1.15)
Social grades ABC1	76.6	82.7	88.8	1.08 (1.04, 1.12)	1.07 (1.04, 1.10)
Social grades C2DE	69.2	73.8	78.4	1.07 (1.00, 1.14)	1.06 (1.00, 1.13)
Children in household	77.1	83.3	88.7	1.08 (1.02, 1.15)	1.07 (1.02, 1.12)
No children in household	72.8	78.8	84.5	1.08 (1.04, 1.13)	1.07 (1.04, 1.11)
Current smoking	68.2	70.4	76.4	1.03 (0.93, 1.15)	1.09 (0.99, 1.19)
Former smoking	72.1	79.1	86.9	1.10 (1.03, 1.17)	1.10 (1.04, 1.16)
Never smoking	76.2	82.9	87.1	1.09 (1.04, 1.14)	1.05 (1.01, 1.09)
E-cigarette use	71.0	68.6	76.6	0.97 (0.84, 1.11)	1.12 (1.00, 1.25)
No e-cigarette use	74.1	81.4	86.4	1.10 (1.06, 1.13)	1.06 (1.03, 1.09)

Adjusted analyses

Table S7: Differences in level of support between years in Great Britain by subgroups and years (unweighted N=6541, table data weighted), in form of prevalence ratios, adjusted using the most common category of the respective covariates (age: 35+; gender: women; social grade: ABC1; children in the household: no; smoking status: never smoking; e-cigarette use: no).

	**Adjusted prevalence ratios (95% CI)**	
	2021/2022	2022/20223
**Ban the sale of cigarettes and tobacco products to everyone born after a certain year**		
Age 18-34	1.22 (1.03, 1.46)	1.17 (1.00, 1.36)
Age 35+	1.32 (1.20, 1.45)	1.10 (1.01, 1.19)
Women	1.29 (1.15, 1.45)	1.09 (0.99, 1.21)
Men	1.28 (1.14, 1.43)	1.13 (1.03, 1.24)
Social grades ABC1	1.35 (1.22, 1.49)	1.15 (1.06, 1.24)
Social grades C2DE	1.20 (1.05, 1.37)	1.08 (0.95, 1.23)
Children in household	1.30 (1.12, 1.50)	1.10 (0.98, 1.24)
No children in household	1.27 (1.15, 1.41)	1.11 (1.03, 1.21)
Current smoking	1.41 (1.02, 1.96)	1.31 (1.01, 1.71)
Former smoking	1.33 (1.11, 1.59)	1.19 (1.02, 1.39)
Never smoking	1.29 (1.16, 1.43)	1.07 (0.97, 1.17)
E-cigarette use	1.10 (0.77, 1.56)	1.29 (0.99, 1.69)
No e-cigarette use	1.30 (1.20, 1.42)	1.10 (1.02, 1.18)
**Raising the legal age of sale of cigarettes and tobacco from 18 to 21**		
Age 18-34	1.19 (1.00, 1.42)	1.06 (0.91, 1.23)
Age 35+	1.15 (1.04, 1.26)	1.12 (1.03, 1.21)
Women	1.08 (1.00, 1.18)	1.15 (1.07, 1.24)
Men	1.24 (1.14, 1.36)	1.05 (0.98, 1.13)
Social grades ABC1	1.19 (1.10, 1.27)	1.12 (1.06, 1.19)
Social grades C2DE	1.11 (1.01, 1.22)	1.07 (0.98, 1.17)
Children in household	1.29 (1.16, 1.43)	1.05 (0.97, 1.14)
No children in household	1.09 (1.02, 1.17)	1.11 (1.05, 1.18)
Current smoking	1.23 (1.02, 1.49)	0.98 (0.83, 1.16)
Former smoking	1.10 (0.97, 1.25)	1.20 (1.07, 1.34)
Never smoking	1.17 (1.09, 1.27)	1.10 (1.03, 1.18)
E-cigarette use	1.06 (0.85, 1.33)	1.03 (0.84, 1.26)
No e-cigarette use	1.16 (1.09, 1.24)	1.10 (1.05, 1.16)
**Making e-cigarettes available on prescription as a stop-smoking aid for adult smokers**		
Age 18-34	0.91 (0.76, 1.08)	0.96 (0.82, 1.12)
Age 35+	0.97 (0.88, 1.06)	1.12 (1.03, 1.21)
Women	0.99 (0.88, 1.11)	1.06 (0.95, 1.19)
Men	0.91 (0.82, 1.02)	1.08 (0.97, 1.20)
Social grades ABC1	0.93 (0.84, 1.03)	1.13 (1.03, 1.24)
Social grades C2DE	0.97 (0.85, 1.11)	1.00 (0.87, 1.15)
Children in household	0.85 (0.71, 1.01)	1.14 (0.97, 1.34)
No children in household	0.99 (0.90, 1.09)	1.05 (0.96, 1.15)
Current smoking	0.97 (0.76, 1.25)	1.05 (0.84, 1.32)
Former smoking	0.93 (0.79, 1.09)	1.02 (0.87, 1.19)
Never smoking	0.95 (0.85, 1.06)	1.10 (1.00, 1.22)
E-cigarette use	1.00 (0.83, 1.21)	1.09 (0.92, 1.29)
No e-cigarette use	0.94 (0.86, 1.03)	1.10 (1.04, 1.16)
**Restricting e-cigarette advertising to prevent uptake by young people**		
Age 18-34	1.03 (0.86, 1.23)	1.06 (0.91, 1.24)
Age 35+	1.07 (0.97, 1.18)	1.04 (0.96, 1.13)
Women	1.09 (1.04, 1.13)	1.04 (1.00, 1.07)
Men	1.04 (0.99, 1.09)	1.07 (1.03, 1.11)
Social grades ABC1	1.07 (1.03, 1.11)	1.07 (1.04, 1.10)
Social grades C2DE	1.06 (1.00, 1.13)	1.07 (1.00, 1.15)
Children in household	1.06 (1.01, 1.11)	1.04 (1.00, 1.08)
No children in household	1.05 (1.02, 1.09)	1.04 (1.02, 1.07)
Current smoking	1.06 (0.97, 1.16)	1.04 (0.97, 1.11)
Former smoking	1.04 (0.98, 1.11)	1.09 (1.04, 1.14)
Never smoking	1.08 (1.03, 1.12)	1.03 (1.00, 1.06)
E-cigarette use	0.97 (0.88, 1.08)	0.95 (0.82, 1.10)
No e-cigarette use	1.07 (1.04, 1.10)	1.09 (1.01, 1.19)

Table S8: Differences in level of support between years in Great Britain by subgroups and years (unweighted, N=6541), in form of prevalence ratios, adjusted using the most common category of the respective covariates (age: 35+; gender: women; social grade: ABC1; children in the household: no; smoking status: never smoking; e-cigarette use: no).

	**Adjusted prevalence ratios (95% CI)**	
	2021/2022	2022/20223
**Ban the sale of cigarettes and tobacco products to everyone born after a certain year**		
Age 18-34	1.26 (1.08, 1.48)	1.18 (1.03, 1.35)
Age 35+	1.31 (1.20, 1.43)	1.10 (1.03, 1.18)
Women	1.30 (1.17, 1.45)	1.09 (1.00, 1.19)
Men	1.29 (1.16, 1.43)	1.14 (1.05, 1.24)
Social grades ABC1	1.34 (1.22, 1.47)	1.16 (1.07, 1.24)
Social grades C2DE	1.21 (1.07, 1.36)	1.04 (0.93, 1.17)
Children in household	1.34 (1.18, 1.52)	1.10 (0.99, 1.21)
No children in household	1.26 (1.15, 1.38)	1.12 (1.04, 1.21)
Current smoking	1.46 (1.10, 1.95)	1.17 (0.92, 1.47)
Former smoking	1.33 (1.13, 1.57)	1.17 (1.02, 1.34)
Never smoking	1.29 (1.24, 1.34)	1.10 (1.07, 1.14)
E-cigarette use	1.10 (0.81, 1.50)	1.24 (0.98, 1.58)
No e-cigarette use	1.31 (1.21, 1.42)	1.11 (1.04, 1.19)
**Raising the legal age of sale of cigarettes and tobacco from 18 to 21**		
Age 18-34	1.20 (1.07, 1.35)	1.05 (0.95, 1.16)
Age 35+	1.12 (1.06, 1.19)	1.12 (1.07, 1.18)
Women	1.10 (1.03, 1.19)	1.13 (1.06, 1.21)
Men	1.19 (1.10, 1.29)	1.08 (1.01, 1.16)
Social grades ABC1	1.16 (1.09, 1.24)	1.12 (1.06, 1.18)
Social grades C2DE	1.09 (1.00, 1.19)	1.07 (0.99, 1.16)
Children in household	1.29 (1.17, 1.41)	1.05 (0.98, 1.13)
No children in household	1.08 (1.02, 1.15)	1.12 (1.06, 1.17)
Current smoking	1.18 (1.00, 1.40)	1.00 (0.86, 1.16)
Former smoking	1.11 (1.00, 1.24)	1.17 (1.06, 1.29)
Never smoking	1.16 (1.11, 1.20)	1.10 (1.06, 1.15)
E-cigarette use	1.01 (0.84, 1.22)	1.11 (0.96, 1.29)
No e-cigarette use	1.15 (1.09, 1.22)	1.10 (1.05, 1.15)
**Making e-cigarettes available on prescription as a stop-smoking aid for adult smokers**		
Age 18-34	0.90 (0.78, 1.04)	0.98 (0.85, 1.12)
Age 35+	0.96 (0.88. 1.05)	1.12 (1.03, 1.21)
Women	0.98 (0.89, 1.10)	1.07 (0.97, 1.18)
Men	0.91 (0.82, 1.00)	1.09 (0.99, 1.19)
Social grades ABC1	0.94 (0.86, 1.04)	1.11 (1.02, 1.20)
Social grades C2DE	0.94 (0.83, 1.07)	1.03 (0.91, 1.17)
Children in household	0.90 (0.78, 1.05)	1.10 (0.96, 1.27)
No children in household	0.96 (0.87, 1.05)	1.08 (0.99, 1.16)
Current smoking	1.00 (0.80, 1.25)	1.02 (0.83, 1.25)
Former smoking	0.91 (0.79, 1.05)	1.06 (0.92, 1.21)
Never smoking	0.95 (0.91, 0.98)	1.11 (1.08, 1.14)
E-cigarette use	0.94 (0.80, 1.11)	1.00 (0.87, 1.14)
No e-cigarette use	0.95 (0.87, 1.02)	1.09 (1.02, 1.18)
**Restricting e-cigarette advertising to prevent uptake by young people**		
Age 18-34	1.04 (0.98, 1.10)	1.07 (1.02, 1.12)
Age 35+	1.07 (1.04, 1.10)	1.05 (1.02, 1.07)
Women	1.09 (1.05, 1.13)	1.03 (1.00, 1.06)
Men	1.05 (1.00, 1.09)	1.08 (1.04, 1.11)
Social grades ABC1	1.07 (1.04, 1.11)	1.06 (1.04, 1.09)
Social grades C2DE	1.06 (1.01, 1.12)	1.05 (1.00, 1.10)
Children in household	1.06 (1.01, 1.11)	1.05 (1.01, 1.08)
No children in household	1.06 (1.03, 1.09)	1.05 (1.02, 1.07)
Current smoking	1.03 (0.95, 1.12)	1.05 (0.99, 1.12)
Former smoking	1.08 (1.02, 1.14)	1.07 (1.03, 1.12)
Never smoking	1.07 (1.03, 1.11)	1.04 (1.01, 1.07)
E-cigarette use	0.98 (0.89, 1.08)	1.07 (0.99, 1.15)
No e-cigarette use	1.07 (1.04, 1.10)	1.05 (1.03, 1.07)

References

1. AddictO. Tobacco smoker. 27 September 2022 2020. https://addictovocab.org/ADDICTO:0000405 (accessed 19 September 2023).
2. AddictO. Former tobacco smoker. 16 February 2022 2020. https://addictovocab.org/ADDICTO:0000713 (accessed 19 September 2023).
3. AddictO. Never smoker. 16 February 2022 2020. https://addictovocab.org/ADDICTO:0000779 (accessed 19 September 2023).
4. AddictO. E-cigarette use. 6 January 2021 2020. https://addictovocab.org/ADDICTO:0000664 (accessed 19 September 2023).
5. Collis D. Social grade: A classification tool–Bite sized through piece. London, United Kingdom, 2009.
